# Correlation of ATP Citrate Lyase and Acetyl CoA Levels with Trichothecene Production in *Fusarium* *graminearum*

**DOI:** 10.3390/toxins5112258

**Published:** 2013-11-20

**Authors:** Naoko Sakamoto, Rie Tsuyuki, Tomoya Yoshinari, Jermnak Usuma, Tomohiro Furukawa, Hiromichi Nagasawa, Shohei Sakuda

**Affiliations:** 1 Department of Applied Biological Chemistry, University of Tokyo, 1-1-1 Yayoi, Bunkyo-ku, Tokyo 113-8657, Japan; E-Mails: m.n.s@pony.ocn.ne.jp (N.S.); rie_yoshinari@takasago.com (R.T.); plenamioga@hotmail.com (J.U.); mountainxtc@live.jp (T.F.); anagahi@mail.ecc.u-tokyo.ac.jp (H.N.); 2 National Institute of Health Sciences, 1-18-1 Kamiyoga, Setagaya-ku, Tokyo 158-8501, Japan; E-Mail: t-yoshinari@nihs.go.jp

**Keywords:** ATP citrate lyase, precocene II, acetyl CoA, trichothecene production, cobalt chloride, sucrose, *Fusarium graminearum*

## Abstract

Thecorrelation of ATP citrate lyase (ACL) and acetyl CoA levels with trichothecene production in *Fusarium graminearum* was investigated using an inhibitor (precocene II) and an enhancer (cobalt chloride) of trichothecene production by changing carbon sources in liquid medium. When precocene II (30 µM) was added to inhibit trichothecene production in a trichothecene high-production medium containing sucrose, ACL expression was reduced and *ACL* mRNA level as well as acetyl CoA amount in the fungal cells were reduced to the levels observed in a trichothecene trace-production medium containing glucose or fructose. The *ACL* mRNA level was greatly increased by addition of cobalt chloride in the trichothecene high-production medium, but not in the trichothecene trace-production medium. Levels were reduced to those level in the trichothecene trace-production medium by addition of precocene II (300 µM) together with cobalt chloride. These results suggest that ACL expression is activated in the presence of sucrose and that acetyl CoA produced by the increased ALC level may be used for trichothecene production in the fungus. These findings also suggest that sucrose is important for the action of cobalt chloride in activating trichothecene production and that precocene II may affect a step down-stream of the target of cobalt chloride.

## 1. Introduction

*Fusarium graminearum* is a worldwide predominant plant pathogen that causes Fusarium head blight of wheat and other grain cereals and produces trichothecene mycotoxins in infected grains. Contamination of important cereal crops by trichothecenes, mainly deoxynivalenol and nivalenol, is a serious human and livestock health concern and also has the potential to cause drastic economic consequences [1]. Presently, the use of fungicide is the most effective method for controlling contamination, but inhibition of fungal growth may lead to the spread of resistant fungal strains [2]. Therefore, it is critical to develop other effective means for controlling trichothecene contamination. To determine the optimal target for developing an effective method, a better understanding of the basic regulatory mechanisms for trichothecene production in the fungus is required.

Trichothecenes are biosynthesized from farnesyl pyrophosphate, which is produced *via* the mevalonate pathway. Mevalonate, the key intermediate in the pathway, is biosynthesized from three acetyl CoA molecules. A number of *Tri* genes are responsible for trichothecene biosynthesis from farnesyl pyrophosphate [3]. In *F. graminearum*, *Tri6* encodes a key regulatory protein for trichothecene biosynthesis [4,5]. The expression of *Tri* genes encoding trichothecene biosynthetic enzymes is under the positive control of TRI6. TRI6 also up-regulates the expression of genes encoding enzymes involved in the mevalonate pathway [6]. Therefore, the overall biosynthetic pathway from acetyl CoA to trichothecenes is activated by TRI6. However, the upstream events that lead to TRI6 expression have not yet been clarified. 

Supply of acetyl CoA to the biosynthetic pathway may be necessary for trichothecene production, but the mechanism by which acetyl CoA is supplied is not clear. Three pathways, in which acetyl CoA synthetase, carnitine acetyltransferase, or ATP citrate lyase (ACL) is a key enzyme, are known to produce acetyl CoA in the fungal cytosol [7]. Occurrence of ACL and carnitine acetyltransferase activity was shown to be spread widely in filamentous fungi [8]. It has been shown that each subunit of ACL of *F. graminearum* is encoded by two genes (*ACL1* and *ACL2*) and deletion of *ACL1* and/or *ACL2* results in reduction of trichothecene production in the fungus [9]. It also has been shown that two carnitine acetyltransferases (CAT1 and CAT2) are present in the fungus and deletion of *CAT1* decreases trichothecene production [10]. In our proteome analysis studies on the mode of action of precocene II, a trichothecene production inhibitor, we found that ACL2 expression was reduced by precocene II. In a microarray experiment with the *Tri6* deletion mutant, *ACL* mRNA levels were not affected by the deletion, suggesting that its expression is not under the control of TRI6 [5]. Although acetyl CoA levels are thought to be a key factor for trichothecene production, the relationship of ACL expression levels or acetyl CoA amounts with trichothecene production has not been investigated. Therefore, in this study we investigated the relationship using factors that affect trichothecene production.

Carbon source is an important factor for trichothecene production in *F. graminearum* [11]. Sucrose and some oligosaccharides containing a sucrose moiety are necessary for active production of trichothecene. Other sugars, such as glucose or fructose, do not induce the production. The molecular mechanism of sucrose for induction of trichothecene production is not clear. Precocene II and cobalt chloride are known as a specific inhibitor and enhancer for trichothecene production of *F. graminearum*, respectively [12,13]. Precocene II reduced the mRNA levels of *Tri6*, whereas cobalt chloride enhanced levels. The target molecules of precocene II and cobalt chloride have not yet been elucidated.

In this paper, we describe the results of the proteome analysis and experiments designed to investigate the correlation of the ACL expression level and acetyl CoA amount with trichothecene production in *F. graminearum* using precocene II and/or cobalt chloride under different carbon sources.

## 2. Results and Discussion

### 2.1. Effects of Precocene II on ACL Expression

*Fusarium graminearum* MAFF101551 accumulates 3-acetyldeoxynivalenol (3-ADON) in its culture supernatant in SYEP liquid medium containing sucrose as a carbon source. The production of 3-ADON by the strain begins 2 days after cultivation and reaches a plateau at 4 days of cultivation in the medium [12]. Precocene II, a constituent of essential oils, almost completely inhibited 3-ADON production of strain MAFF101551 at a concentration of 30 µM without affecting fungal growth and ergosterol production. Precocene II may affect a regulatory mechanism leading to expression of TRI6 in the trichothecene production pathway in the fungus. To investigate the mode of action of precocene II, proteome analysis by two-dimensional differential gel electrophoresis (2D-DIGE) was performed [14,15]. 

**Figure 1:**
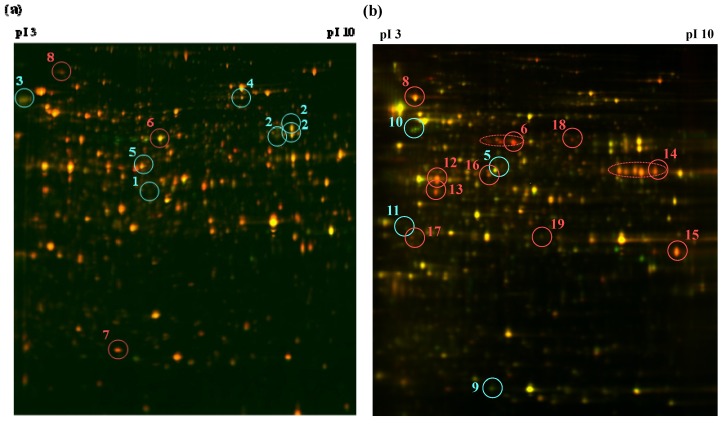
A two-dimensional differential gel electrophoresis (2D-DIGE) gel image of *F. graminearum* proteome after precocene II treatment for 2 days (**a**) or 4 days (**b**). Green, red, and yellow spots indicate proteins with decreased, increased, and unchanged abundance levels, respectively. Selected proteins (>1.2 and <1.2 change in abundance; *p* < 0.05; *n* = 3) are numbered.

Strain MAFF101551 was incubated without or with precocene II (30 µM) for 2 or 4 days and the proteins were extracted from the cells. Control and precocene II-treated samples were labeled with Cy3 (green) and Cy5 (red), respectively, and combined. The mixture was separated by two-dimensional gel electrophoresis followed by fluorescent imaging (Figure 1). Green spots (decreased with precocene II treatment) and red spots (increased with precocene II treatment), showing changes in abundance greater than 1.2-fold, were clearly observed on the gels. Proteins in the spots were identified by MALDI-TOFMS analysis after in-gel tryptic digestion and by MASCOT research (Table 1). Among the identified proteins, diphosphomevalonate decarboxylase (spot 11 on the sample gel from day 4), an enzyme involved in the mevalonate pathway, was identified as a protein that had decreased expression after precocene II treatment. Since expression of diphosphomevalonate decarboxylase is promoted by TRI6, inhibition of TRI6 expression by precocene II may lead to a decrease of the protein expression level. We also focused on the decrease of ACL (ACL2 encoded by FGSG_06039.3, [8]) expression (spot 5 on sample gels from days 2 and 4) because ACL was a key enzyme for acetyl CoA supply into the cytosol as described above. The relationship of the other identified proteins listed in Table 1 with precocene II’s function is currently under investigation.

**Table 1:** Analysis of the effect of precocene II by 2D-DIGE.

Spot	Protein	Fold change ^a^		pI ^b^	kDa	Expect	Matched peptides	Sequence coverage (%)
		2 days	4 days					
1	Formamidase	−1.43	-	5.6	44.2	3.1 × 10^−^^10^	9	29
2	Serine carboxypeptidase	−1.53	-	6.5	59.7	0.0019	5	10
3	Serine carboxypeptidase	−1.54	-	5.1	63.6	0.029	6	13
4	Peroxidase catalase 2	−1.43	-	5.8	81.3	3.1× 10^−^^11^	11	17
5	ATP citrate lyase	−1.27	−1.59	5.5	53.3	5.0 × 10^−^^7^	8	26
6	Pyruvate decarboxylase	+1.60	+4.29	5.6	63.5	2.0 × 10^−^^13^	12	34
7	Glycolipid transfer protein	+1.33	-	5.7	22.4	6.0 × 10^−^^5^	5	30
8	Cell division cycle protein 48	+1.35	+2.85	4.9	90.5	0.00087	6	11
9	Superoxide dismutase	-	−2.27	5.6	27.7	4.0 × 10^−^^7^	6	29
10	Kinesin heavy chain	-	−2.56	5.7	104.1	19	4	6
11	Diphosphomevalonate decarboxylase	-	−1.77	5.3	41.0	2.2	3	7
12	Enolase	-	+2.32	5.0	47.5	1.0 × 10^−^^10^	10	35
13	Carboxypeptidase S1	-	+2.18	5.5	52.3	6.3 × 10^−^^7^	8	28
14	Acid phosphatase	-	+1.85	6.6	47.8	0.15	4	10
15	Alcohol dehydrogenase 1	-	+2.39	7.6	42.0	0.0061	5	19
16	Aldehyde dehydrogenase	-	+1.60	5.4	53.9	1.0 × 10^−^^12^	11	32
17	40S ribosomal protein 50	-	+2.34	4.8	31.8	3.1 × 10^−^^10^	8	30
18	Pyruvate kinase	-	+2.78	5.8	60.0	4.8 × 10^−^^5^	7	13
19	UDP glucose epimerase	-	+1.69	5.8	42.0	0.0051	5	20

^a^ Normalized spot intensity: precocene II treated *vs.* untreated cells (average of triplicate gels from each of the three independent cultures). ^b^ Theoretical pI (isoelectric point) was calculated from amino acid sequence.

### 2.2. Effects of Carbon Sources on Acetyl CoA Amount and *ACL2* mRNA Level

**Figure 2:**
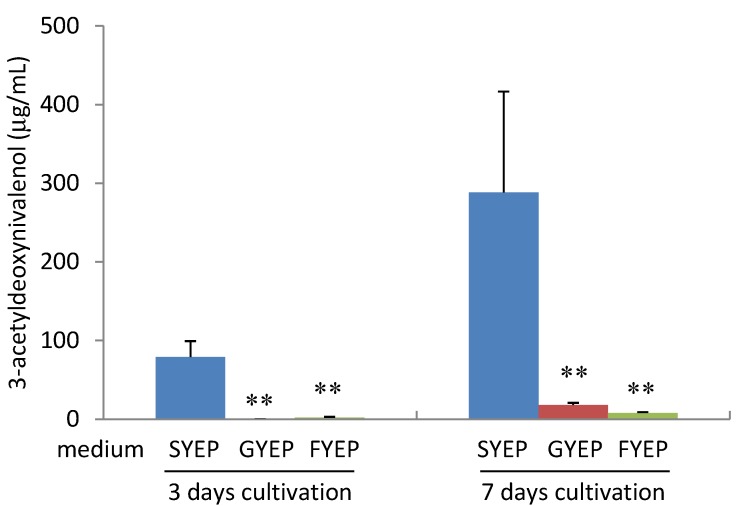
Effects of carbon sources on 3-acetyldeoxynivalenol production by *F. graminearum*. *n* = 3, ** *p* < 0.01, *vs.* control.

**Figure 3:**
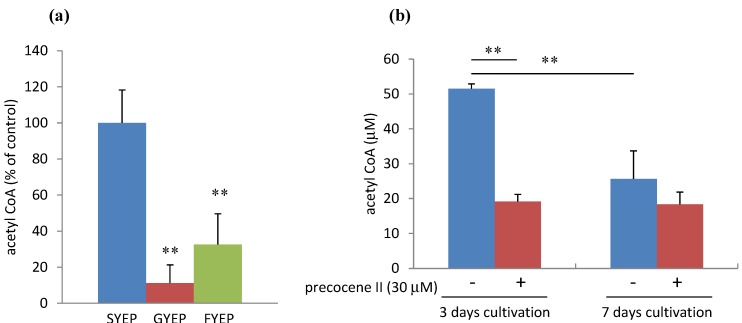
Effects of carbon sources (**a**) and precocene II in SYEP medium (**b**) on acetyl CoA amount. *n* = 3, ** *p* < 0.01, *vs.* control.

**Figure 4:**
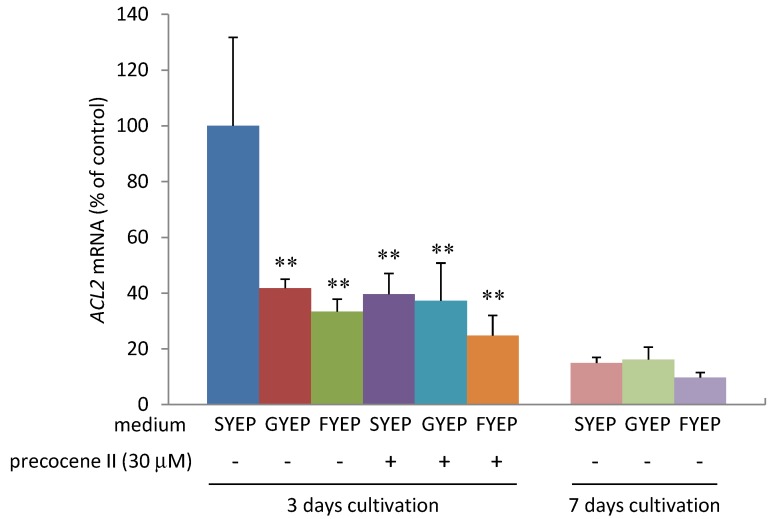
Effects of carbon sources and precocene II on *ACL2* mRNA level. *n* = 4 (3 days cultivation), *n* = 3 (7 days cultivation), ** *p* < 0.01, *vs.* control.

As reported by Jiao *et al.* [11], strain MAFF101551 produced a very small amount of 3-ADON in GYEP or FYEP liquid medium containing glucose or fructose as a carbon source instead of sucrose in SYEP medium at 3 and 7 days cultivation (Figure 2). We analyzed the levels of acetyl CoA and *ACL2* mRNA in the fungal cells cultured in these three media. Analysis of acetyl CoA amount was performed using the method by Ruijter *et al.* with some modification [16]. The acetyl CoA amount in the fungal cells cultured in SYEP liquid medium was much higher than that in GYEP or FYEP medium at 3 days of cultivation (Figure 3). A decrease of the acetyl CoA amount was observed after 7 days of cultivation in SYEP medium. The fungal cells cultured in SYEP medium showed the highest *ACL2* mRNA level among the three media at 3 days of cultivation (Figure 4). However, after 7 days of cultivation, the *ACL2* mRNA level was nearly the same among all three conditions. These results suggest that the acetyl CoA amount and *ACL2* mRNA level correlate with trichothecene production of *F. graminearum* and that sucrose is a key factor for activating *ACL2* transcription and acetyl CoA production. 

### 2.3. Effects of Precocene II on Acetyl CoA Amount and *ACL2* mRNA Level

The acetyl CoA amount in the fungal cells cultured in SYEP medium was strongly reduced by addition of 30 µM precocene II at 3 days of cultivation (Figure 3), but it was not significantly affected after 7 days of cultivation in SYEP medium. The *ACL2* mRNA level observed in SYEP medium was also reduced to the level detected in GYEP or FYEP by addition of precocene II at 3 days of cultivation (Figure 4). In addition, precocene II did not have a strong effect on the transcription level of *ACL2* in GYEP or FYEP. These results suggest that precocene II may suppress the activation of *ACL2* transcription and acetyl CoA production by sucrose.

### 2.4. Effects of Cobalt Chloride on Acetyl CoA Amount and *ACL2* mRNA Level

**Figure 5:**
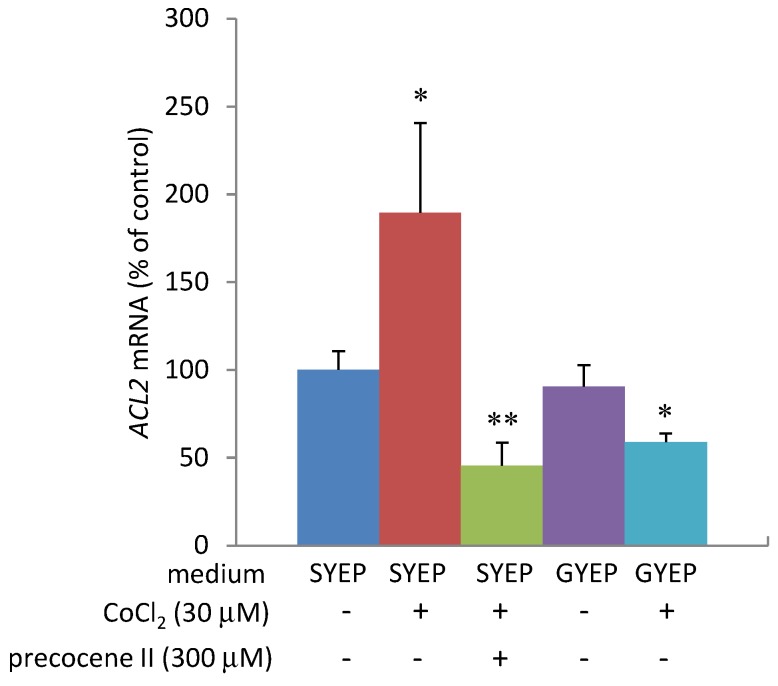
Effects of cobalt chloride on *ACL2* mRNA level. *n* = 4, ** *p* < 0.01, * *p* < 0.05, *vs.* control.

**Figure 6:**
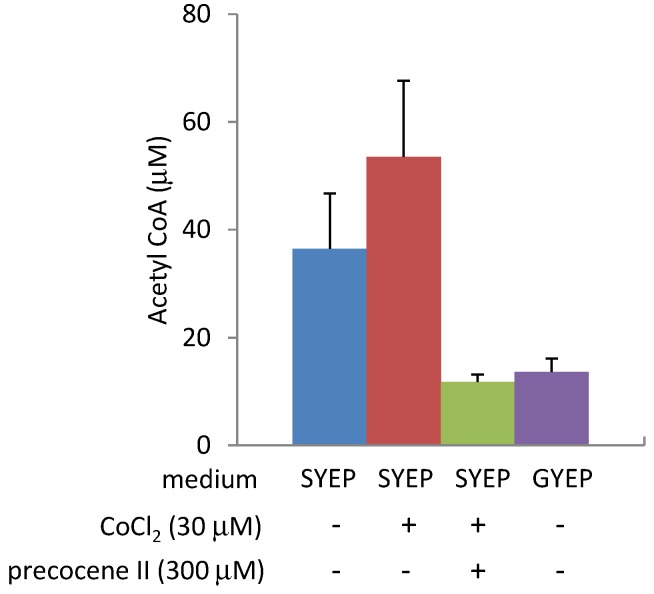
Effects of cobalt chloride on acetyl CoA amount. *n* = 3.

The production of 3-ADON in strain MAFF101551 is known to be dramatically enhanced by addition of 30 µM cobalt chloride in SYEP medium without any effect on fungal mycelial weight and ergosterol amount [13]. Cobalt chloride strongly up-regulated transcription of *Tri6* and genes regulated by TRI6 as well as genes encoding ergosterol biosynthetic enzymes. Higher concentrations (300 µM) of precocene II were necessary to almost completely inhibit 3-ADON production in SYEP medium containing 30 µM cobalt chloride. In the presence of 30 µM of cobalt chloride and 300 µM of precocene II, transcription of *Tri6* was suppressed, but the transcription of ergosterol biosynthetic enzyme genes was still up-regulated [13]. After addition of 30 µM cobalt chloride, the *ACL2* mRNA level in the fungal cells was enhanced in SYEP liquid medium, but not significantly affected in GYEP medium (Figure 5). The acetyl CoA amount in the fungal cells was slightly increased by addition of 30 µM cobalt chloride in SYEP medium, but not in GYEP medium (Figure 6). Co-addition of 300 µM of precocene II into SYEP medium containing 30 µM of cobalt chloride reduced the *ACL2* mRNA level and acetyl CoA amount to those observed in GYEP medium (Figure 5 and Figure 6).

**Figure 7:**
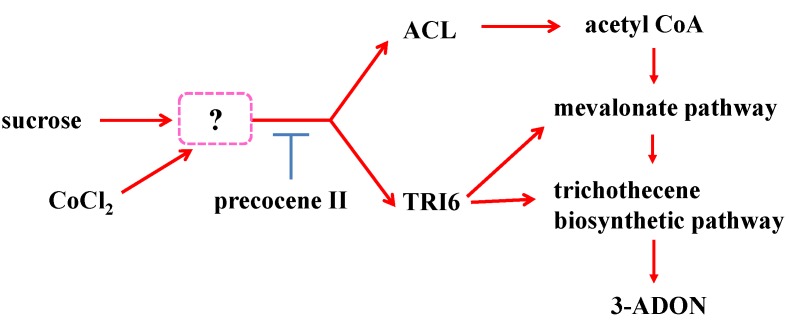
Possible regulatory mechanism of trichothecene production by *F. graminearum*.

Based on our results, we propose a regulatory mechanism for trichothecene production (Figure 7) whereby the expression of ACL and TRI6 is activated in the presence of sucrose, and the acetyl CoA produced by the increased ALC levels may be used for trichothecene production in *F. graminearum*. Sucrose is important for the action of cobalt chloride in activating transcription of *ACL* and *Tri6*, which leads to promotion of trichothecene production. Precocene II may affect a step down-stream of the target of cobalt chloride. *F. graminearum* can produce many secondary metabolites including polyketides and terpens other than trichothecenes [17]. Since acetyl CoA is the key precursor common to biosynthesis of polyketides and terpens produced by the fungus, increasing of acetyl CoA amount may affect their production. Therefore, the results obtained in this study may have a possibility to provide clues for clarifying the regulatory mechanism of production of not only trichothecenes but also other secondary metabolites produced by the fungus. 

## 3. Experimental Section

### 3.1. *F. graminearum* Culture Conditions and Analysis of 3-Acetyldeoxynivalenol

A Japanese isolate strain, *Fusarium graminearum* MAFF101551, described previously [13] was used as a 3-acetyldeoxynivalenol producer. A spore suspension of the strain was prepared using carnation leaf agar medium. After cultivation of the strain on the medium at 28 °C for 10 d, the leaf was rinsed in 20% glycerol aqueous solution and the mixture was filtered with miracloth to obtain a filtrate containing spores, which was stored at −80 °C and used as a spore suspension. Liquid medium (5 mL) of SYEP (sucrose 5%, yeast extract 0.1%, polypeptone 0.1%), GYEP (d-glucose 5%, yeast extract 0.1%, polypeptone 0.1%), or FYEP (d-fructose 5%, yeast extract 0.1%, polypeptone 0.1%) was put into test tubes (1.6 cm × 18 cm) and autoclaved. Autoclaved aqueous CoCl_2_ solution (5 μL) and/or methanolic precocene II solution (15 μL) was added to the medium. Each tube was inoculated with a spore suspension of the strain (1 × 10^5^ spores/tube) and then incubated with continuous shaking (300 rpm) at 26.5 °C for 2–7 d. The resulting culture broth was filtered to obtain the mycelia and filtrate. The filtrate (1 mL) was extracted with 200 µL of ethyl acetate, the ethyl acetate solution was evaporated to dryness, and the obtained residue was dissolved in 200 µL of 10% acetonitrile in water, which was subjected to LC/MS analysis using a 2695 HPLC system (Waters, Milford, MA, USA) equipped with a 150 mm × 2 mm i.d. Capcell-Pak C_18_ column eluted with a gradient of 10%–80% acetonitrile in water containing 10 mM ammonium acetate in 20 min. The flow rate was 0.2 mL/min and the retention time of 3-acetyldeoxynivalenol was 9.4 min. MS analysis was done with a micromassZQ (Waters) by ESI, in positive ion mode; spray chamber parameters: source temperature, 120 °C; desolvation temperature, 350 °C; cone, 30 V; desolvation gas, 600 L/h; cone gas, 50 L/h; capillary voltage, 2800 V. MS ions were monitored in single-ion recording mode using the extracted ion *m/z* 339 (M + H)^+^.

### 3.2. 2D-DIGE Analysis

A spore suspension of the strain MAFF101551was inoculated into SYEP medium (5 mL) in a test tube with or without precocene II (30 μM), and incubated with continuous shaking (300 rpm) at 26.5 °C for 2 d or 4 d. After incubation, mycelia were harvested and lyophilized. The dried mycelia were ground in a mortar with a pestle in liquid nitrogen, and incubated in CelLytic Y Yeast Cell Lysis/Extraction Reagent (600 µL, Sigma-Aldrich, St. Louis, MO, USA) containing 1 M DTT solution (2 µL) and phosphatase inhibitor cocktail (2 µL, Sigma-Aldrich) for 30 min at room temperature to extract total proteins. After centrifugation of the mixture at 15,000 *g* for 10 min at 4 °C, the supernatant was mixed with acetone (800 µL) and incubated for 1 h at −80 °C. The obtained precipitates were collected by centrifugation, washed with ethanol, and dissolved in 2D-DIGE lysis buffer (2 M thiourea, 7 M urea, 4% (*w*/*v*) CHAPS, 30 mM Tris-HCl, pH 8.5). Total protein contents were determined by the Bradford assay using BSA as a standard.Main text paragraph 

Precocene II-treated and control protein samples were quantified and labeled with NHS-Cy2, -Cy3 and -Cy5 (GE Healthcare, Buckinghamshire, UK). Protein (50 μg) taken from precocene II-treated or control samples were minimally labeled with 160 pmol of Cy3 or Cy5 in triplicate. An equal pool of precocene II-treated and control samples were labeled with Cy2 (3.2 pmol/μg protein) and run as a standard on all gels to aid in spot matching and cross-gel quantitative analysis. Protein labeling was performed on ice in the dark for 30 min. Reactions were quenched by 10 min incubation with a 20-fold molar excess of free lysine. The labeled samples were mixed and carrier Pharmalyte (pH 3–10, GE Healthcare) was added to a final concentration of 2%.

The mixture was separated by isoelectric focusing in the first dimension using 24 cm pH 3–10 NL (nonlinear) strip (GE Healthcare) and by SDS-PAGE in the second dimension using 10% SDS-PAGE gel bonded to low-fluorescence glass plates. Gels were run in Ettan 6 gel tanks at 400 mA per gel at 20 °C until the dye front had run off the bottom.

After 2DE, gels were scanned using a Typhoon™ 9400 variable mode imager (GE Healthcare) and ImageQuant software (GE Healthcare). The photomultiplier tube voltage was adjusted for each dye channel for preliminary low-resolution scans to give maximum pixel values within 5%–10% for each channel and below saturation, prior to the acquisition of 100 μm high-resolution images. Images were cropped and analyzed using DeCyder™ V5.0 (GE Healthcare). Ratios of spot intensities detected in precocene II-treated or control samples to those of corresponding spots in standard sample were calibrated and obtained values were averaged across triplicates for each experimental condition. Statistical analysis was performed to pick spots matching across all images, displaying a ≥1.2 average-fold increase or decrease in abundance between drug-treated and control samples and with *p* values < 0.05 (Student’s *t*-test). 

### 3.3. In-Gel Digestion and Protein Identification by Mass Spectrometry

Visible spots were excised from the CBB R250 (Nacalai tesque, Kyoto, Japan) -stained gels and transferred into microcentrifuge tubes. Excised fragments were washed successively with water, 25 mM NH_4_HCO_3_, acetonitrile/25 mM NH_4_HCO_3_ (1:1) and acetonitrile. Destained gel fragments were dried under vacuum with a centrifugal evaporator. Tryptic digestion was performed overnight at 37 °C using 10 µL of 10 µg/mL trypsin (Roche, Basel, Switzerland) in 50 mM NH_4_HCO_3_, pH 7.8. The resulting tryptic fragments were extracted twice with 100 µL of acetonitrile/water (3:2) containing 0.1% TFA, in an ultrasonic bath for 15 min. The supernatants were concentrated to 10 µL in a centrifugal evaporator and passed through a Zip-Tip™ C18 (Millipore, Bedford, MA, USA). The adsorbed peptides were eluted with 2.0 µL of acetonitrile/water (3:2) containing 0.1% TFA and 1% α-cyano-4-hydroxycinnamic acid. The eluent was loaded onto a mass spectrometer sample plate. Mass spectra were acquired on a Voyager-DE STR MALDI-TOF mass spectrometer (Applied Biosystems, Foster City, CA, USA) operated in the reflectron-delayed extraction mode. Spectra were internally calibrated using trypsin autodigestion products. MASCOT (Matrix Science, Boston, MA, USA) was used to search databases. The expect value, number of matched peptides and percentage of sequence coverage for each identified protein are listed in Table 1.

### 3.4. Analysis of Acetyl CoA

The strain MAFF101551 was incubated in liquid medium (5 mL), and the mycelia were obtained and frozen by liquid nitrogen. The frozen mycelia were incubated for 30 min at −45 °C with a solution of methanol (6 mL) and chloroform (10 mL) containing 3,4-dimethoxyaniline (1 µM) as the internal standard. After adding an aqueous 5 mM triethanolamine solution (4 mL) to the mixture, the mixture was incubated for 10 min at −45 °C and then shaken for 30 min at room temperature. After centrifuging the mixture at 5000 *g* for 5 min at −10 °C, the upper layer was transferred to a 50 mL tube. The remaining lower layer was mixed with 5 mM triethanolamine (2 mL) and shaken. After centrifuging the mixture at 5000 *g* for 5 min at −10 °C, the upper layer was transferred into the 50 mL tube in which the first upper layer has been collected. The pooled upper layers were shaken with diethyl ether (15 mL) and centrifuged at 5000 *g* for 5 min at −10 °C to obtain the lower layer. After the solution was filtered and evaporated, the obtained residue was dissolved in 200 µL of 25% methanol in water, which was subjected to LC/MS analysis under the same conditions as 3-acetyldeoxynivalenol was analyzed above mentioned except for the following ones. Isocratic elution by solvent A (5 mM hexylamine in water whose pH was adjusted to 6.3 by acetic acid) from 0 to 2 min and then gradient elution by changing the ratio of solvent A and solvent B (90% methanol in water containing 10 mM ammonium acetate whose pH was adjusted to 8.5 by ammonia water) from 100:0 to 0:100 from 2–50 min were used for the HPLC elution. The retention time of acetyl CoA was 33.2 min. MS ions were monitored in single-ion recording mode using the extracted ion *m/z* 810 (M + H)^+^.

### 3.5. RT-PCR Analysis

The strain MAFF101551 was incubated in liquid medium (5 mL) and mycelial cake was harvested by filtration and lyophilized. Total RNA was extracted using a TRIzol plus RNA Purification Kit (Invitrogen, Carlsbad, CA, USA) according to the manufacturer’s protocol. First-strand cDNA was prepared using the SuperScript III First Strand Synthesis System (Invitrogen) with random hexamer primers, according to the protocol. The cDNA derived from 0.005 μg of total RNA was used as a template. Real-time quantitative RT-PCR was carried out using the SYBR Green Master Mix (Applied Biosystems, Foster City, CA, USA), in a final volume of 25 μL for each reaction, and an ABI PRISM 7300 thermal cycler (Applied Biosystems). Two-step PCR conditions were as follows: after an initial incubation at 95 °C for 10 min, 40 cycles of 95 °C for 15 s and 60 °C for 1 min were performed. The PCR primers for each gene were as follows: *ACL2* 5'-CGCCAACTACGGCGAGTAC-3' and 5'-AGTTCGGGCGTAGTGGTAAGTC-3': *β-tubulin* (control gene) 5'-CCTGACCTGCTCTGCCATCT-3' and 5'-TGGTCCTCAACCTCCTTCATG-3'. The amount of each mRNA was normalized to the amount of *β-tubulin* mRNA in each sample.

### 3.6. Data Analysis

Data are presented as the mean ± SD. Differences between groups were assessed with one-way ANOVA followed by Dunnett’s test. Values of *p* < 0.05 were considered to be significant. 

## 4. Conclusions

The relationship of ATP citrate lyase and acetyl CoA levels to trichothecene production in *F. graminearum* was clearly observed in the trichothecene-production medium containing sucrose, which suggested that up-regulation of expression of ATP citrate lyase by sucrose is a key event for inducing the trichothecene biosynthesis. 
